# A Case of Chronic Myelomonocytic Leukemia With Recurrent Skin Involvement

**DOI:** 10.7759/cureus.75193

**Published:** 2024-12-05

**Authors:** Eren Arslan Davulcu, Suat Hilal Akı

**Affiliations:** 1 Hematology Clinic, University of Health Sciences Bakırkoy Dr. Sadi Konuk Training and Research Hospital, Istanbul, TUR; 2 Pathology Department, Istanbul University-Cerrahpasa, Cerrahpasa Medical Faculty, Istanbul, TUR

**Keywords:** azacytidine, chronic myelomonocytic leukemia (cmml), chronic myeloproliferative neoplasms, hydroxyurea, myelodysplastic syndromes, skin involvement

## Abstract

Chronic myelomonocytic leukemia is a clonal hematopoietic stem cell disorder with both myelodysplastic and myeloproliferative features, leading to a variable clinical presentation. Some types of skin involvement, such as leukemia cutis and blastic plasmacytoid dendritic cell neoplasia, are associated with poor prognosis. This case study describes a 71-year-old male with high-risk CMML, developing pink-purple skin nodules, which regressed with azacitidine and hydroxyurea treatment. Despite recurrence, disease control was achieved without transformation to acute leukemia. This case highlights the need for vigilant monitoring and adaptable treatment strategies in managing CMML with skin involvement.

## Introduction

Chronic myelomonocytic leukemia (CMML) is a clonal hematopoietic stem cell disorder that occupies a unique position within the myelodysplastic/myeloproliferative neoplasia group due to its simultaneous myelodysplastic and myeloproliferative characteristics [1]. This dual nature results in a complex clinical presentation that includes features of both bone marrow failure, typical of myelodysplastic syndromes, and proliferative symptoms such as elevated white blood cell counts, seen in myeloproliferative disorders. Among the myriad manifestations of CMML, skin involvement remains a rare but significant finding, further complicating the clinical picture. The dermatological manifestations of CMML are diverse and have been reported under various classifications in the literature, reflecting the heterogeneous nature of the disease.

One of the aggressive forms that may be encountered in CMML patients is leukemia cutis (LC), where leukemic cells infiltrate the skin, leading to nodular or plaque-like lesions that can vary in color from red to violaceous, and the other is blastic plasmacytoid dendritic cell neoplasia (BPDCN). Both BPDCN and LC are particularly notable for their poor prognosis. Additionally, multicentric reticulohistiocytosis has been reported in CMML patients, presenting with multiple nodules and papules caused by histiocyte accumulation in the skin. This condition is often associated with systemic symptoms and can significantly impact a patient's quality of life. Histiocyte/monocyte infiltration in the skin represents another variant, where abnormal collections of these cells lead to various cutaneous manifestations [2-5]. In addition to histiocytic and monocytic skin infiltrations, skin involvements associated with systemic vasculitis and neutrophilic dermatosis can also be seen in the course of CMML [6].

The molecular mechanisms driving the invasion of histiocytes or leukemic cells into the skin remain poorly understood. Lymphocytes, various chemokine receptors, and specific adhesion molecule receptors might contribute to the migration of these cells into the dermis. 

The variability in skin lesion classification highlights the complex and multifaceted nature of CMML with skin involvement. Each type of lesion not only signifies a different pathological process but also necessitates distinct diagnostic and therapeutic approaches. The skin involvement in CMML poses challenges in diagnosis and treatment, emphasizing the need for heightened awareness and comprehensive clinical evaluation to manage this multifaceted disease effectively.

## Case presentation

**Figure 1:**
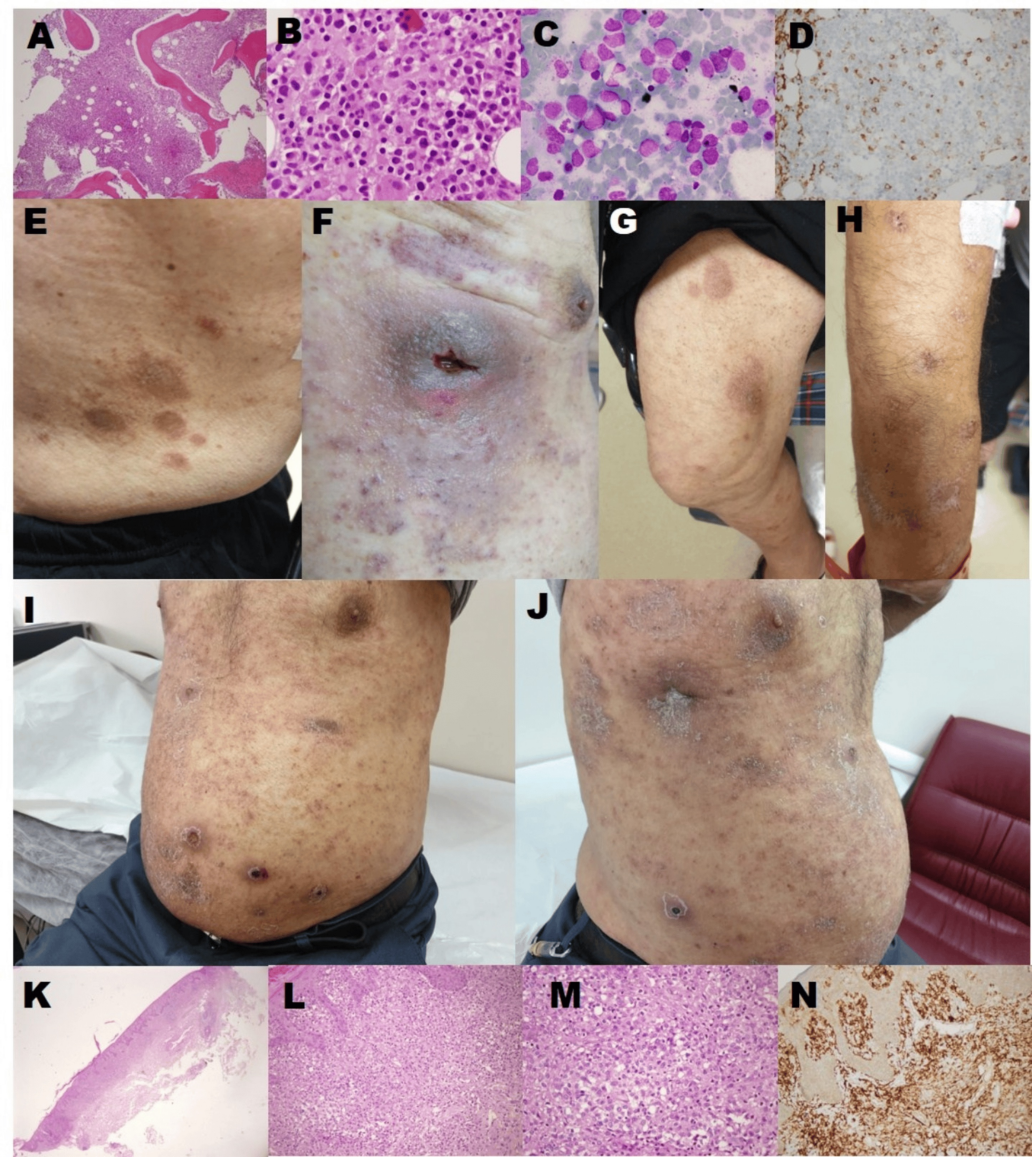
Clinical and pathological findings Initial bone marrow biopsy revealed hypercellularity, myeloid hyperplasia, dysmegakaryopoiesis, and 6% blasts (A, B, C) and CD 14 positivity (D). The first episode of skin involvement demonstrated pink-purple nodular lesions in his trunk and extremities (E, F, G H). Second episode of skin involvement (I, J). Skin biopsy during the second episode of skin lesions taken from a lesion on the trunk showed reactive lymphocytes and cells with large cytoplasm, thin chromatin, and a partially large nucleus in the epidermis (K, L, M), and CD 14 positivity in the skin (N).

While a 71-year-old male patient was being examined for monocytosis, anemia, and hepatosplenomegaly (liver 190 mm, spleen 180 mm in abdominal ultrasonography), his tests revealed that leukocyte 41000/mm^3^, neutrophil 29000/mm^3^, monocyte 7840/mm^3^, lymphocyte 2000/mm^3^ hemoglobin 9.5 g/dL, MCV 95 fL, platelet 75000/mm^3^, C-reactive protein 20 mg/L (normal level <5), serum uric acid 13 mg/dL (normal range 3.4-7 mg/dL), ferritin 197 µg/L (normal range 23-336 µg/L), and lactate dehydrogenase 712 U/L (normal range 135-214 U/L). Bone marrow biopsy supported the diagnosis of CMML with hypercellularity (%95), hyperplasia in the myeloid lineage, increased monocytes, uninterrupted maturation, increased focal megakaryopoiesis, dysmegakaryopoiesis, and increased blasts (6%) (Figures 1A-1D).

The disease was classified as myeloproliferative type, CMML-1 [7]. Cytogenetic analysis of the bone marrow disclosed 46, XY. NRAS, SF3B1, and TET2 mutations were detected positive. It was evaluated as a high-risk CMML according to the CPSS-mol classification model [8]. He was considered not to be a candidate for allogeneic stem cell transplantation due to his comorbid diseases, such as advanced congestive heart failure and coronary artery disease. The treatment plan was azacitidine 75 mg/m2 on days 1 to 7.

Just before azacitidine treatment was initiated, pink-purple nodular lesions, the largest of which was up to 5 cm, developed on the legs and trunk (Figures 1E-1H). The lesion on the right side of the thorax turned into an abscess (Figure 1F). A skin biopsy performed with the preliminary diagnosis of LC was not diagnostic due to technical inadequacies. The lesions completely regressed with azacitidine treatment under appropriate antibiotic therapy. Before the fourth course of azacitidine treatment, the same but smaller lesions recurred in similar locations (Figures 1I, 1J).

Meanwhile, the monocyte count increased from 1500 to 5600/mm³, and the neutrophil count from 5600 to 22,000/mm³ without worsening of anemia and thrombocytopenia, an increase in hepatosplenomegaly, or peripheral blasts. When the skin lesions appeared for the second time, the bone marrow biopsy performed with the preliminary diagnosis of transformation into acute leukemia was again compatible with CMML-1. The second skin biopsy, including deeper layers, was obtained from the trunk. It showed reactive lymphocytes and mature monocytes with large cytoplasm, thin chromatin, and a partially large nucleus in the epidermis. These cells were CD68+ and CD14+ but did not show staining with MPO, c-kit, CD34, TdT, CD20, CD30, CD15, and CD123. It was evaluated as monocytic skin infiltration of CMML (Figures 1K-1N). Hydroxyurea 1000 mg/day combined with azacitidine on consecutive days. The patient completed seven courses of azacitidine treatment with a 'clinical benefit' [9]. Skin lesions disappeared with the combination of hydroxyurea and azacitidine. Just before the eighth course, the patient died from respiratory failure due to viral pneumonia.

## Discussion

Studies have classified skin lesions seen during CMML differently [2,3]. These cells are distinguished according to their histopathological and immunophenotypic characteristics. Blastic histopathologic features such as high nucleus/cytoplasm ratio, loose chromatin, and high mitosis have different immunophenotypes depending on the characteristics of the infiltrating tumor [4]. The cells in our patient's skin biopsy were evaluated as non-blastic because they were mature-looking monocytes and CD34 negative. It has been reported in the literature that non-malignant skin lesions are seen in the course of CMML [4,5]. It is postulated that such skin involvements do not have a poor prognosis as in malignant skin infiltrates [5]. In our patient, monocytosis increased with the appearance of skin lesions, but no blastic transformation was detected in either the bone marrow or the skin. Clinical findings were controlled by starting treatment at the first appearance and then intensifying it with hydroxyurea.

There is literature information that hydroxyurea antagonizes the effects of azacitidine and decitabine. It is suggested that this antagonism can be overcome by sequential treatment with hydroxyurea and hypomethylating agents [10]. However, for our patient, since there was no other treatment option due to his age and clinical condition and proliferative symptoms became prominent, hydroxyurea was added sequentially for cytoreduction [11]. Since skin involvement in CMML is very diverse, the treatment approach is different for each type of involvement. For example, patients with LC or BPDCN need to receive more intensive treatments, including allogeneic stem cell transplantation. There are currently no specific treatment approaches for managing non-blastic monocytic infiltrates, as seen in our patient.

Next-generation sequencing (NGS) assays play a crucial role in the diagnosis and prognosis of CMML. Most CMML patients will exhibit detectable somatic mutations in genes involved in epigenetic regulation, splicing, signaling, and transcription. These mutations have a role in the pathogenesis of CMML, and some of them have prognostic significance. The most current approach to CMML risk scoring is CPSS-mol, which is based on these molecular data [7]. According to this scoring system, mutations in ASXL1, NRAS, RUNX1, and SETBP1 worsen prognostic risk. NRAS, SF3B1, and TET2 mutations were detected in our patient. Among these, TET2 mutations have not been shown to independently impact either overall or leukemia-free survival [12,13]. Similarly, SF3B1 mutation does not have a poor prognostic effect, yet it is associated with a favorable leukemia-free survival [14]. Oncogenic RAS pathway mutations, including NRAS, are associated with a myeloproliferative neoplasm-like phenotype and associated with transformation from CMML to acute leukemia [15]. Clonal RAS pathway mutations are known to correlate with proliferative CMML [16]. The proliferative type CMML picture in our patient and perhaps the extramedullary involvement may be related to the involvement of the RAS pathway.

Although it is difficult to make clear predictions in terms of prognosis since he has only received seven courses of treatment, the disease was under control clinically, and there was no blastic transformation. NGS could not be performed on the patient's skin biopsy. Finding results similar to those detected in bone marrow by NGS would be important to support the existence of a clonal relationship between CMML and skin lesions.

## Conclusions

LC and BPDCN in CMML are crucial due to their impact on prognosis and the potential need for more intensive treatments. In this case, CMML exhibited skin involvement without typical progression markers such as increased blast percentage or organomegaly, though there was a slight rise in monocyte and neutrophil counts. This pattern suggests that CMML with skin involvement can be effectively managed by initiating the treatment and, in some scenarios, intensifying it with a combination of available drugs. The patient's experience underscores the importance of vigilant monitoring and adaptive treatment strategies in managing CMML. NGS assays are essential for diagnosing and prognosing CMML. In our patient, the detected NRAS mutation aligns with a myeloproliferative phenotype and may contribute to the proliferative CMML and possible extramedullary involvement. Performing NGS in biopsies from extramedullary involvement, such as skin, is of great importance in terms of demonstrating clonality both in the bone marrow and other tissues. This case illustrates that, despite the complexities and challenges posed by CMML with skin involvement, appropriate and timely adjustments in treatment can lead to successful disease management. Enhanced awareness and comprehensive evaluation are essential for optimizing outcomes in patients with this multifaceted disease.
